# Distributed ledger technology based robust access control and real-time synchronization for consumer electronics

**DOI:** 10.7717/peerj-cs.566

**Published:** 2021-06-01

**Authors:** Mohd Majid Akhtar, Mohammad Zubair Khan, Mohd Abdul Ahad, Abdulfattah Noorwali, Danish Raza Rizvi, Chinmay Chakraborty

**Affiliations:** 1 Department of Computer Engineering, Jamia Millia Islamia University, New Delhi, India; 2 Department of Computer Science, College of Computer Science and Engineering, Taibah University, Medina, Saudi Arabia; 3 Department of Computer Science and Engineering, Jamia Hamdard University, New Delhi, India; 4 Electrical Engineering Department, Umm Al-Qura University, Makkah, Saudi Arabia; 5 Department of Electronics and Communication Engineering, Birla Institute of Technology, Ranchi, Jharkhand, India

**Keywords:** DLT, Blockchain, Consumer electronics, IoT, IOTA, Access control

## Abstract

**Background:**

Consumer electronics or daily use home appliances are the basic necessity of every household. With the adoption of IoT in consumer electronics, this industry is set to rise exponentially. In recent times, the demand for consumer electronics rises amidst the pandemic due to a paradigm shift from in-office culture to work from home. Despite intelligent IoT devices, smart home configuration, and appliances at our disposal, the rudimentary client-server architecture fails to provide facilities like full access control of data and devices, transparency, secured communication, and synchronization between multiple devices, etc. to the users.

**Methods:**

To overcome these limitations, Blockchain technology has been adopted in recent years, however, it has its own set of limitations in its widespread implementation. Hence, we propose a methodology using the IOTA platform, a distributed ledger technology (DLT) for secured communication between consumer electronics devices and appliances.

**Results:**

The implementation provides access control, interoperability, data storage, and management with an exploratory insight towards a decentralized micro-payment use-case between Electric cars and charging stations.

## Introduction

The term “consumer electronics” is used for the electronic devices envisioned for daily usage in a typical household setup. It may include devices for “entertainment”, “utilities”, “recreation”, “communication” etc. With the rapid advancement of technology in recent years, legacy Consumer Electronics (CE) devices are getting smarter and are very commonly seen in every household. Every household has some kind of smart devices (TV, Fan, Bulb, washing machines, dishwashers, etc.) which are capable of doing much more than the legacy devices available a decade ago. With the Internet of Things (IoT) based embedded intelligence, these smart devices can sense the environment and nearby conditions and has the capabilities to act autonomously based on the sensed information. IoT and Artificial Intelligence have played a very crucial role in the transition of typical CE devices to smart consumer electronic devices. Intelligence is incorporated in consumer electronics to provide ease of usage and convenience to the users. The ability to remotely control and manage these devices through mobile Apps or other wearable devices enhances the user experience and convenience. This is the reason that there is an exponential rise in the adoption of smart devices across domains including consumer electronics, medical equipment’s, fitness and personalized devices, etc. Although the transition of classical consumer electronic devices to smart devices seems to be very easy, several inherent issues and challenges need to be resolved for its success. Some of these challenging factors are (Eskicioglu & Delp, 2001; Heiser, 2009):

• Compatibility

Compatibility is very crucial while converting the legacy of consumer electronics to smart devices. Most of the legacy devices do not have the provision for changing the circuits for embedding IoT based sensors. Other compatibility issues include power wattage, resistance, durability, etc. Secondly, most of the smart devices are proprietary which makes them incompatible with other devices of different make and models.

• Interoperability

The ability of devices to work on more than one platform is known as interoperability. The traditional consumer electronic devices are generally not interoperable, thus inducing intelligence in these devices is a complex task that involves adding some external interfaces in these devices for smooth transitions and interoperability.

• Device and Information security

Device and information security are the prime concern of consumer electronic devices. The data captured by the sensors embedded in these devices is very crucial for taking various autonomous decisions. If this data is not secured, there can be adverse consequences leading to financial and or personal harm to the individual or organizations.

• Access control

Since the smart electronic devices may be operated by multiple users, it is very important to identify the user access level of each user and to grant only a specific set of permissions to operate the devices. For example, a normal user must not be granted access to the security codes and passwords of the devices.

The other form of access control is data access control. Data access control is normally achieved by using traditional client/server architecture which remains a bottleneck to hack and various data privacy breaches. Hence, with advance decentralized approach, data present on distributed ledger will be under user control with full access rights to it.

• Data ownership

The questions like who owns what data and what time is highly crucial. In case of any mishaps, there must be well-defined provisions identifying the ownership of the data in the transition to fix the responsibility of the individuals (users, manufacturers, retailers, etc.) or organizations.

Other problems with CE Devices and Appliances are:

• Lack of Standardization for machine-to-machine models
• No full Access Control over device and data
• Real-Time Synchronization delay and issues
• Lack of Secured channel for data communication between CE appliances
• Heavy reliance on centralized architectures and server

Another very important factor with smart devices is the vast amount of big data generated by these devices. The complex and varied nature of data from thousands of sensors embedded within the smart devices makes it much more difficult to manage by conventional data management systems. Few challenges of big data include: heterogeneity, scalability, variety, exponential rate of data generation, veracity etc. The smart devices management systems must have provisions to handle such challenges only then it can be successful (Ahad & Biswas, 2017; Ahad & Biswas, 2019). Data Clustering and classification are other challenges that needs to be resolved for providing effective and personalized services to the end users (Casalino, Castellano & Mencar, 2018; Casalino, Castellano & Mencar, 2019). Similarly, the authors in Xiong et al. (2020) discussed an incentive-based scheme for sending messages in order to improve delivery rate and reduce the frequent dropping of messages. In Du et al. (2019), the authors proposed DST-ICRL model for traffic flow prediction in urban settlements in Beijing and New York areas. The empirical evaluation was conducted which shows significant improvement in the prediction rate as compared to existing methods. In Qiu et al. (2020), the authors proposed an adversarial attack against NID Systems which resulted in a higher attack success rate with minimal modification of 0.005% of bytes in the malicious packet.

We have used a decentralized research approach to solve two genuine problem of consumer electronics i.e., access control and real time synchronization between devices. The research method involves *IOTA platform* which aims to be standard for Internet of Things (IoT) economy. With the help of following this research method, we were able to establish a decentralized way of communication between different modules of electronic items. Our results are based on simulator program using IOTA libraries.

### Major Contributions

The major contributions of this paper are:

(1) Distributed Ledger Technology (DLT) based innovative solution for Consumer Electronics has been proposed.

(2) Research gaps in existing Blockchain-based approaches and IOTA in terms of scalability, real-time synchronization, and access control are discussed.

(3) It provides IOTA core communication protocols stack among devices and nodes.

(4) In this paper, we presented a decentralized micro-payment use-case between an electric car and charging stations using DLT based solution that enables future smart devices economy. The proposed approach excels in the performance parameters like scalability, energy consumption, confirmed transaction per second, real-time synchronization, access control, transaction cost, real-time IoT applicability as well as interoperability/modularity concerning other existing state-of-the-art of research.

### Related works

This section presents the background and existing works and objectives for the need of decentralized solutions for consumer electronics.

### Blockchain for consumer electronics

Lee (2018) Presented blockchain and IoT can exist together in a complementary fashion. Several researchers explored the use of Blockchain technology in mobile phones with the help of complex and advanced smart contracts for the application of fingerprint login systems, healthcare data sharing, etc.

An innovative proposed architecture based on a private Ethereum blockchain network running in docker containers were discussed in Putra et al. (2020). They used the network multiple parameters to dynamically calculate the trust and reputation score for each node to achieve access control accordingly.

An exhaustive comparison is done between Proof of Work (PoW) and Proof of Stake for IoT applications feasibility in Akhtar & Rizvi (2020a) and Akhtar & Rizvi (2020b). PoW and PoS are two known consensus algorithms that are required to establish truth in the distributed network. PoW make extensive use of hardware for harnessing hash power to solve a complex puzzle while the latter is mechanism of picking a miner based on staking value and other complex calculations. However, Akhtar & Rizvi (2020a) and Akhtar & Rizvi (2020b) displayed the side of Ethereum blockchain scalability issues and how Hyperledger fabric and composer can precede Ethereum in supply chain use cases.

### Realtime synchronization issues in blockchain for automotive sector

Ethereum blockchain smart contract has been used for multiple use cases in the context of automotive. These applications range from recording driving patterns of the driver using the Global Positioning System for better decision making while insurance claims, recording the mileage reading and odometer readings for fraud prevention (Cebe et al., 2018), storing and securely sharing the vehicle telematics data to mixing blockchain technology with vehicle-to-vehicle communication allowing them to gain blockchain-based trust points under reward-based mechanism (Kang et al., 2018). However, a basic limitation of such solutions is smart contract re-deployment. Smart contracts cannot be changed once deployed. This hinders the production level application since a change in the architecture will nullify the previous smart contract deployed and the on-boarded data it had in it. Data migration from old smart contracts to new contracts is yet not available in Ethereum. However, this can be achieved using proxy contracts acting as a middleware. Hence, we need a modular architecture and not a rigid framework like Ethereum. Scalability issues persist in the Ethereum based application with low transaction per second that hinders the real-time synchronization between devices. Transaction fees can be avoided if using private Ethereum networks.

### Access control issues in blockchain and IoT

Muhammad Salek Ali & Antonelli (2017) Used the Interplanetary File system as storage with Ethereum for storing and accessing IoT devices data. Huh, Cho & Kim (2017) Presented three ‘smart contracts’ for policy control mechanism providing the synchronization between energy reading meter and another device used as a controller for air conditioners. This system is better but not scalable; also transaction fees are applied to each transaction. A better approach on the previous method was adopted with better transaction per second in Novo (2018). Bhushan et al. (2020) displayed how Blockchain can play role in smart cities and smart home applications. Multiple architectures are reviewed and integration trends for sustainable city development were discussed in Ahad et al. (2020) and Lalit et al. (2020). Furthermore (Saxena, Bhushan & Ahad, 2021) provided an extensive review on blockchain technology as a preferred measure to secure applications specific IoT ecosystem.

It was noticed that all the architectures of blockchain lacked the very essence from the point of real-time effectiveness to sustainable and cost-efficient transactions. Blockchain solves this issue primarily but induces new a set of limitations in its implementation. Hence, we propose a methodology using the IOTA platform, a distributed ledger technology for secured communication between consumer electronics devices and appliances. The implementation and experimental work were carried out to show access control, interoperability, data storage, and management with an exploratory insight towards a decentralized micro-payment use-case between Electric cars and charging stations.

## Materials & Methods

This section describes the blockchain-based proposed methodology for consumer electronics.

**Figure 1:**
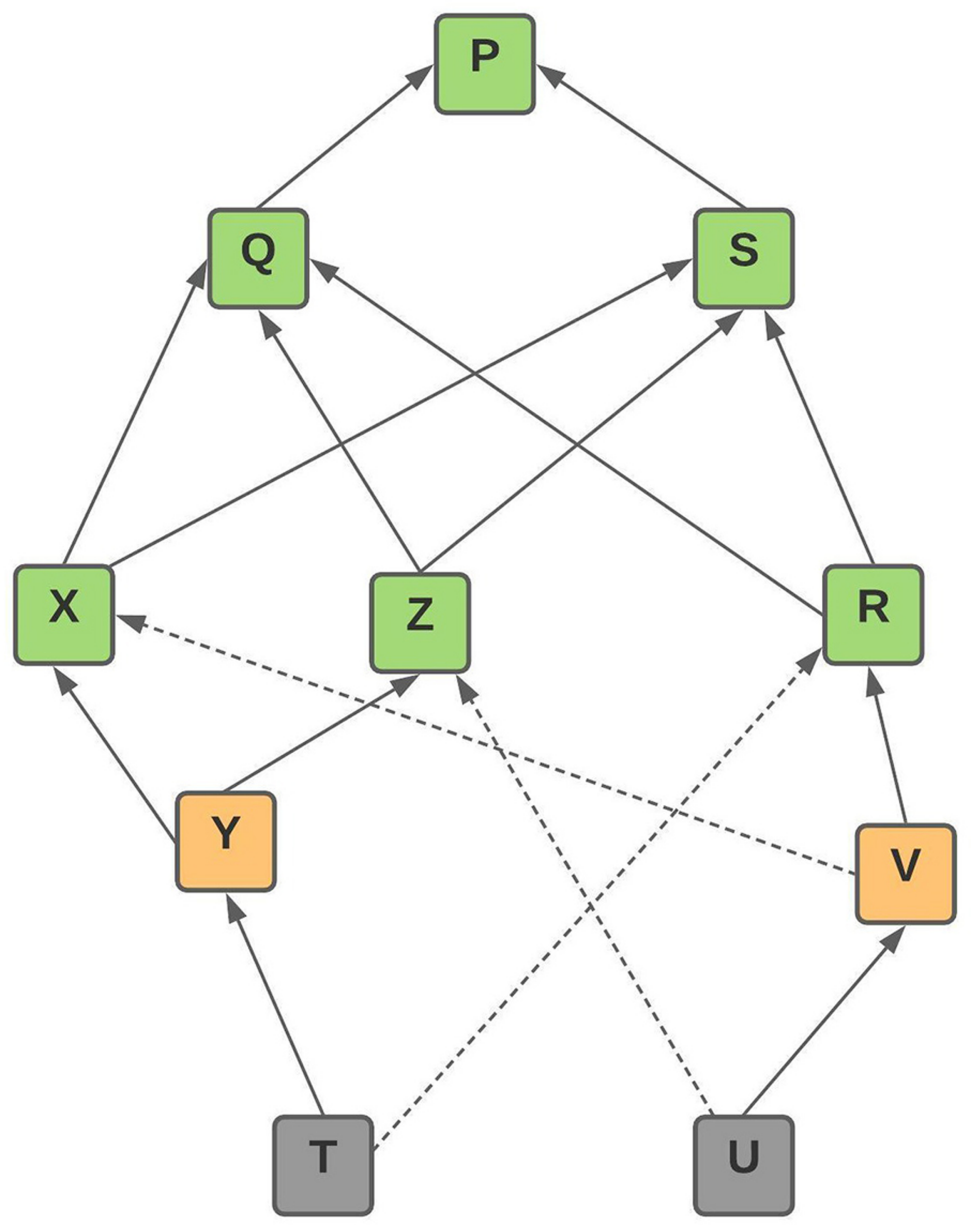
IOTA tangle directed acyclic graph structure.

### Origin of IOTA

In the proposed architecture, we have exploited the use of a distributed ledger technology (DLT). DLT aids secured storage of data into distributed databases without central party maintaining it. It uses advance cryptography to store transactions or records permanently. In this manuscript, we have used IOTA Network (Popov, 2018), a DLT platform that works differently concerning any other blockchain architectures offering more advantages. In 2015, it was founded by Dominik Scheiner, Sergey Ivancheglo, and the team raising $0.5 Million (approx. 1337 bitcoins) from their ‘Initial Coin Offering’. However, the initial project was supposed to be ‘JINN’, ‘next level ternary chips’ that was chosen to process on ternary values instead of binary values. However, multiple setbacks followed the development of JINN chips and the project is overall delayed with no deadline yet.

Characteristics of IOTA Network:

• It is a miner-free as well as the fee-less environment. However, while submitting a transaction, a low energy-intensive ‘PoW’ is done and each transaction has to refer to two previous transactions as shown in Fig. 1.
• Have provision for Public, Private and Restricted architecture.
• Fully decentralized platform.
• It is proven to be quantum resilient because of the involvement of post-quantum era cryptography such as Winternitz One Time Signature.
• Unlike any other Blockchain architectures, Directed Acyclic Graph is the core implemented data structure in IOTA called the ‘Tangle’ as shown in Fig. 1. Here, orange represents ‘unconfirmed transaction’; green represents ‘confirmed transaction’ and grey represent the newest transaction known as ‘tip’. Tips are the newest transaction in the network that has not been referenced by other transactions. In Fig. 1, tip ‘T’ and ‘U’ are the newest transaction added to the network. A tip without any reference for a longer time will never be referenced by transaction as the network only chooses the latest tips for validation at any stage.

To generate a new transaction, each IoT device has to perform three tasks:

• Sign the newly generated transaction in advance before sending the transaction to the Tangle with a unique private key (a combination of seed and the index number).
• Using the Uniform Random Tip Selection (URTS) algorithm, two previous transactions are selected and validated. Each new transaction has to refer to two previous (not very old) transactions. The rule for linking with two previous transactions is to choose two transactions in such a way that they are not very old according to timestamp. Older transactions are not preferred while the linking processes to prevent issue of double spending. Hence, two previously latest transactions are picked according to Uniform Random Tip Selection (URTS) algorithm automatically. This is how IOTA Network progresses. As more and more transactions will come, every new transaction will have some other transaction that will validate it in the future. This increases the scalability of the network even if the number of consumer electronics devices increases on the network.
• A small amount of ‘PoW’ is required to process based on the difficulty defined in Minimum Weight Magnitude (MWM).

### The architecture of core protocols of IOTA used in our approach

**Figure 2:**
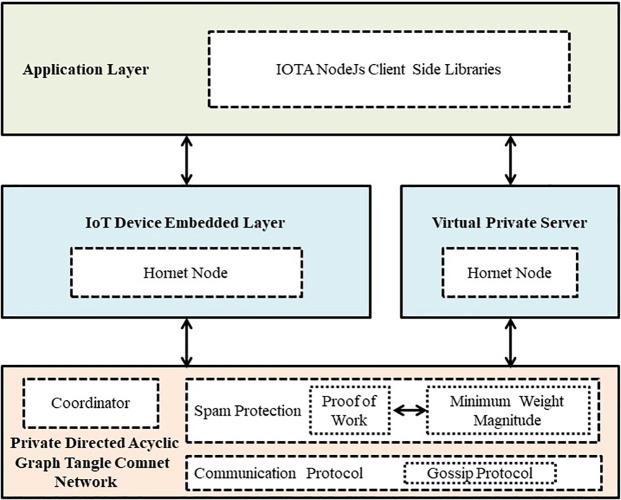
Architecture of core protocols in IOTA private network.

The core protocols in IOTA Private Network are shown in Fig. 2.

### Layer 1: private directed acyclic graph tangle comnet

IOTA offers various functionalities and one such is a *Private Tangle*. A private tangle is an IOTA network of known nodes. Private *Comnet* (Community Network) is the official testnet of IOTA that works similar to the Mainnet except you run the *Coordinator* often known as the ‘Coo’ that is solely responsible for issuing the milestones. Milestones are analogous to *block number* present in bitcoin blockchain which responds to the correctness of ledger state. To prevent private tangle network from spam attacks or distributed denial of service attacks, we have a mechanism for ‘PoW’ which is not as energy exhaustive as in the case of Ethereum and Bitcoin network. Here the difficulty level for ‘PoW’ is decided by the admin specified Minimum Weight Magnitude (MWM). MWM is the trailing number of zeroes in *trits* at the end of the transaction hash. We have kept the MWM same as the default Comnet MWM i.e., 10. This means that the number of ‘0’ trits has to be more than 10 or at least equal to 10 as MWM specified. In summary, MWM specify the amount of work done for submitting the transaction (data) to the Tangle. After validation of the valid MWM, data is accepted to the network and propagated to all the connected nodes using the *Gossip protocol*.

### Layer 2: IoT device and VPS node embedded layer

We need lite node that can smoothly run on the low-power client devices. *Hornet* is one such node software that can interact with *Comnet* (Community Network) private network.

Hornet is the official lite node from IOTA foundation which can run under compute and storage constraints environments. We configured Hornet node on Ubuntu 18.04 (VPS) and Raspberry pi 3. This embedded layer is responsible for acting as a *node URL* that lets us send or receive data from the IOTA Tangle and interact with the Comnet Private Network.

### Layer 3: application layer

For the *application layer* and building strong front end applications, we have used the IOTA client-side libraries in JavaScript. These libraries majorly include *‘mam.js’*, *‘@iota/core’,* and *‘iota.lib.js’*. *‘mam.js’* also known as Masked Authenticated Messaging is used for sending and receiving data to the IOTA Tangle. This package allows three types of data channels for privacy and access control such as Private, Public, and Restricted mode. These masked authenticated message data channels work as a linked list where current data is referenced to the next data using an identifier called *‘root’* and *‘sideKey’*.

### Proposed architecture for sensor equipped smart home application

**Figure 3:**
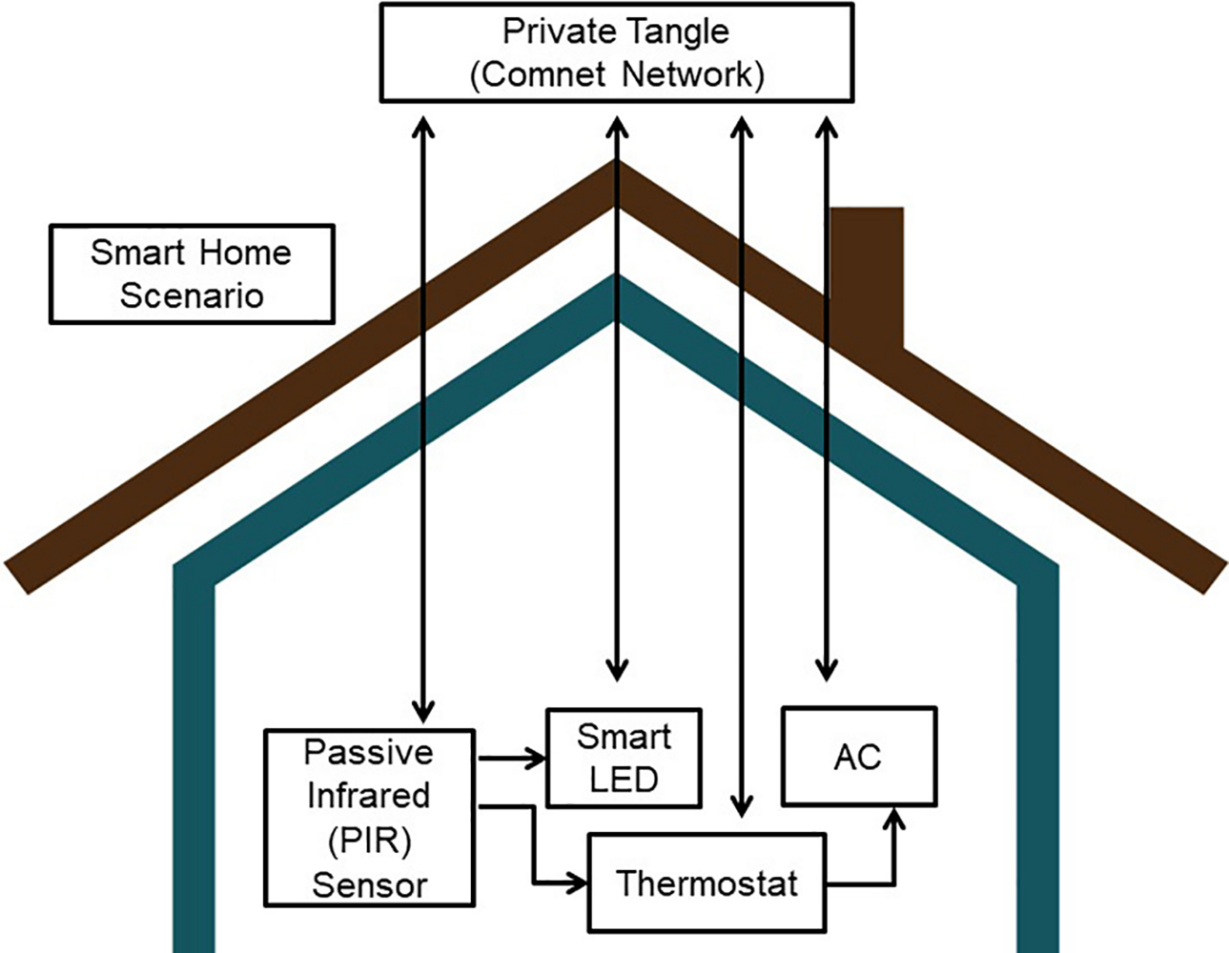
Architecture of smart home application.

**Figure 4:**
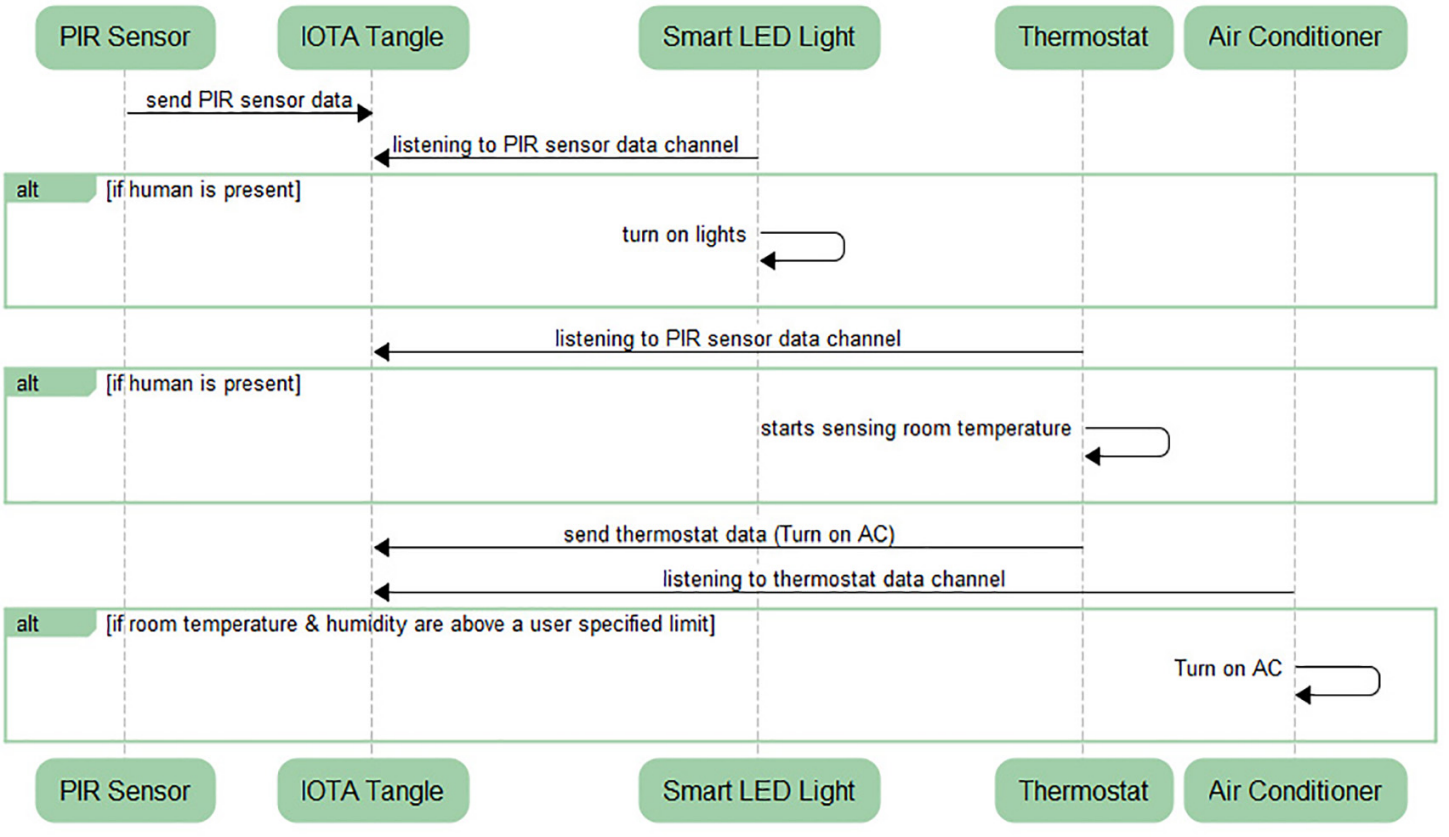
Sequence diagram for IoT devices communication and synchronization.

Figure 3, shows the Smart Home application architecture where devices such as Passive Infrared (PIR) sensor for human detection, smart LED light, thermostat and smart Air Conditioner (AC) are taken into account. They all are connected and paired with the private tangle network. Figure 4, briefly explains the synchronization mechanism between all the devices through the medium of Tangle and IOTA network as data storage.

### The process involved in smart home application

In Fig. 4, *PIR Sensor*, responsible for sensing human presence in the room will be continuously sending its sensed data to the *IOTA Tangle* using restricted mode in its own data channel.

• *Smart LED Light* will be listening to PIR sensor data channel analogous to FM radiofrequency. Similarly, thermostat is listening to the PIR sensor data channel using the *root* and *sidekey* provided by PIR Sensor.
• If *human is present*, the PIR sensor will send high signal resulting to 1 (in digital). When this situation will trigger, Smart LED light will get to know the presence of humans through PIR data channel it is listening to and will turn on the lights as an action. Similarly, if a human is present, the thermostat will start sensing the room temperature and humidity.
• *Thermostat* is sending the room temperature data to the IOTA Tangle. On the other hand, *Air conditioner* (AC) is listening to the thermostat data channel.
• If room temperature and humidity are above a user-specified limit, then AC will get turned on as an action.

### Proposed architecture for electric car gas station communication application

Autonomous IoT devices will be communicating with each other soon. We need a reliable technological and application stack for the smooth functioning of the communication. Synchronization delay and other performance issues persist in the traditional system due to heavy reliance on centralized servers acting as a single point of failure. With the decentralized approach, communication and synchronization become proactive due to which devices have continuous, energy-efficient, and secured data stream channels.

**Figure 5:**
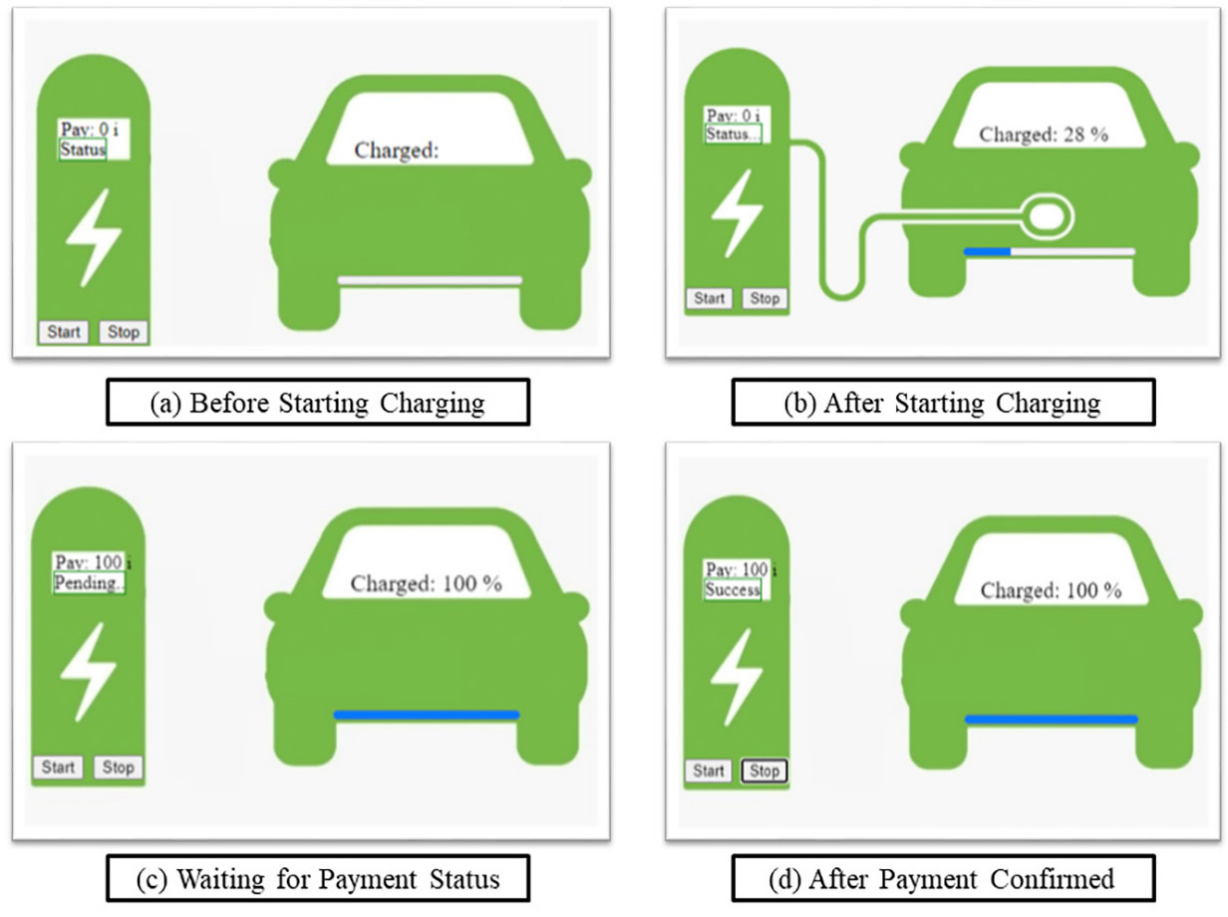
Micro-payment scenario based for electric car - gas charging station. (A) Before starting charging; (B) After starting charging; (C) Waiting for payment status; (D) After payment confirmed.

We have built a proof of concept of how the interaction between the electric car battery and charging stations can take place using ‘IOTA Tokens’, the native currency of IOTA Network, and simulated the solution as web-based software implementation written in JavaScript using *client-side IOTA libraries* and *browerify* for bundling all packages into one JavaScript file. This solution is yet to be tested on real hardware electric car batteries and chargers present at gas stations. Although this solution is proven to be highly modular and not rigid like smart contract of Ethereum. Our architecture is flexible, robust, and scalable complementing the ever-changing needs. In Fig. 5, different stages of interaction between car battery and gas station charger are shown.

In this paper, we have considered 1 IOTA to be deducted per unit of charging from an electric car wallet. These are directly paid to the gas charging wallet through its address. IOTA has 81 *trytes* of addresses. Trytes is similar to bytes in IOTA Network. The payment using IOTA tokens takes approximately 4.5 s due to ‘PoW’ enabled for spam protection.

## Analysis of the Proposed System

This section provides analysis and discussion about the proposed system.

### Address reusability solution

Since, IOTA uses Winternitz One Time Signature, it advises that no address should be used again. This offers security from quantum computing but also induces complexities. One can opined these complexities similar to a lucid example where a user had to change its phone number each time, he/she uses it to call someone. These IOTA *addresses* are generated using a master key known as *Seed*, where funds or tokens can be received. Of course, this needs to be tackled to have an efficient and smooth functioning of the applications built on top of IOTA Network. Some possible solutions and initiatives for address re-usability were Delion API or using IOTA Trinity Wallet.

### Attacks and types of spam on comnet

*Trinity Wallet* is IOTA’s official wallet for storing IOTA Tokens. On February 12, 2020, the Trinity Wallet attack happened due to a third-party dependency from *Moonpay* which resulted in theft of 8.55 *‘Ti’* of IOTA Tokens. This took place due to remote code injection via a dependency, resulting in an illicit version of Moonpay SDK loaded when a user opened its trinity wallet. IOTA has taken measures to fight such theft attacks and iteratively evolving into a mature technology that promises to be revolutionary and innovative for communication between IoT devices.

IOTA Network faces another challenge known as *Spam*. Spamming or Spam is a zero value transaction sent to the IOTA network to speed up the confirmation rate, scalability, and throughput. With spam transactions, the benefit achieved is that the previous two transactions getting confirmed by the incoming new transaction. IOTA network encourages such types of spam events and spammers (those who spam by running ‘PoW’ on their machines) get paid in IOTA’s as a reward for spam. The founder of IOTA stated such spam events act as testing for the network to fix codes, bugs, and giving free research data on how IOTA absorbs such spam attacks organically. These spam tests include parasite chain attack, blowball spam, etc. IOTA Network has incredibly matured than it was a few years back. The confirmation rate is also known as “confirmed transaction per second (CTPS)” in IOTA Network is much higher as compared to other blockchain networks.

## Discussion

Performance parameters like scalability, energy consumption, confirmed transaction per second (CTPS), real-time applicability are necessary while assessing a DLT platform (Akhtar & Rizvi, 2020a; Akhtar & Rizvi, 2020b). Scalability is defined as a system’s nature to handle high request of transaction and changes. If a framework is scalable with upcoming of newer devices and nodes in the network, then the network is said to be scalable. Our model uses remote Proof of Work on behalf of low power devices, hence scalability is ensured. Energy consumption should be as low as possible as most of the devices are low powered battery driven devices. Since, IOTA has no miner, hence, IOTA platform helps in achieving low energy consumption. CTPS is the rate at which transactions are submitted and accepted in the network. IOTA with its tip selection algorithms achieved higher CTPS. In this approach, DDoS attacks are not possible due to decentralized nature. Hence, our work achieves high rate of real time synchronization between different devices and data access control is much easier to achieve. Interoperability is having a modular nature of workflow in the architecture. Our work is built using stack and IOTA libraries that allows our model to be highly modular in nature. The comparisons between our proposed model concerning other five state-of-the-art models based on performance parameters like scalability, energy consumption, CTPS, real-time or critical applicability, etc. are shown in Table 1. Our proposed architecture based on IOTA proves to be scalable and better than the rest of the architectures using Ethereum or Hyperledger umbrella initiatives.

**Table 1:** Comparison between different architectures with respect to our proposed model.

Ref	Scalability	Energy consumption	CTPS	Real time synchronization	Access control	Transaction fees involved	Real time IoT applicability	Interoperability/ modularity
Muhammad Salek Ali & Antonelli (2017)	High	Low	Low	Yes	Yes	Yes	No	No
Huh & C (2017)	Low	High	Low	Yes	Yes	Yes	No	No
Novo (2018)	Low	Low	Low	Yes	Yes	Yes	No	No
Abou-Nassar et al. (2020)	Low	High	High	Yes	Yes	Yes	Yes	No
Proposed approach	High	Low	High	Yes	Yes	No	Yes	Yes

## Conclusion and Future Scope

With our proposed model, smart devices can securely communicate having real-time synchronization. The model will enable trust in the fast-growing consumer electronics industry and manufacturers can rely on the robust and efficient architecture built on the distributed ledger technologies like IOTA. We also aim to mimic our decentralized micro-payment use case between Electric car and charging stations and our smart home architecture from simulated environments to real-world hardware and device implementation as our future work.

The software implementation for the communication between two IoT devices is fully functional. We successfully showcased the true power of decentralization and interaction between two IoT machines is possible without human intervention. Our proposed Smart home application will help to save more energy as the devices are being used only in the presence of humans while maintaining real-time synchronization, security, and access control mechanisms. These devices will even help in the industrial internet of things use cases, where workers don’t have to work in extreme conditions and actions can be performed remotely. Our model can help the industry to run securely and independently using private tangle networks where data and access rights will remain with the owner of the device.

In our future course of the plan, we will focus on “IOTA Access”, an open-source framework for smart devices access control using the defined set of policies. We will follow up with the IOTA Access intrinsic features and protocol and state the maturity of the solution provided by IOTA Foundation.

## Additional Information and Declarations

### Data Availability

The following information was supplied regarding data availability: Data is available at GitHub: https://github.com/mohdmajidakhtar/smartHome_poc.
